# Feasibility of testing antibodies to HIV from filter paper using HIV rapid test kits

**DOI:** 10.1186/1758-2652-13-S4-P147

**Published:** 2010-11-08

**Authors:** A Ganesan, G Thatchinamoorthy, S Saramini

**Affiliations:** 1 The Tamilnadu Dr.MGR Medical University, Department of Experimental Medicine, chennai, India

## 

HIV rapid tests are widely used in resource limited settings. Dried blood spots on filter paper are an easy way to collect and transport blood samples from a remote area to a testing facility at ambient temperatures. Our objective was to evaluate the technical possibility of testing antibodies to HIV from filter paper using HIV rapid tests kits.

Dried blood spots on filter paper were obtained from 408 female sex workers (FSW), 136 Men Sex with Men (MSM) and 50 Intravenous Drug Users (IDU). Antibodies were eluted from DBS disks (punch 6 mm, approximately 5 µl serum) in 200 µl of sterile Phosphate Buffer Saline (PBS) (0.01 M, with 0.1% sodium azide) by agitating overnight at 2-8° C (refrigerator) for minimum 16 hours and tested for anti-HIV antibody using two ELISA (Microlisa, Enzaids). As per the guidelines of the National AIDS Control Organization Sentinel Surveillance of HIV, the reactive samples on the first ELISA are retested using a 2nd ELISA kit. We assessed all the HIV positive and HIV negative DBS samples using 3 HIV rapid tests kits (Combaids, Determine and EIAComb) procedures were followed according to the manufacturer's instructions.

Of the 594 DBS samples, 22 (3.69%) samples were positive for HIV antibodies using ELISA kits. Among HIV positive DBS samples, 3.67% (15/408) were FSW's, 2.95% (4/136) were MSM and 6% (3/50) were IDU's. All 22 DBS were positive in both ELISA kits (Microlisa, Enzaids) and the sensitivity and specificity of both kits were 100%, whereas Determine found 20 HIV positives, Combaids 21 samples Positive and EIAComb found all 22 HIV positive giving sensitivity was 90.91%, 95.45%, and 100% respectively and specificity was 100%.

This study demonstrates and confirms the usefulness and feasibility of the filter paper blood collection method for testing of HIV antibodies. DBS can be used with HIV rapid test devices. Using the latest and advanced techniques applied in rapid test technologies, DBS may provide a unique way to conduct sero epidemiological surveys in resource limited settings.

